# Cognitive control over memory – individual differences in memory performance for emotional and neutral material

**DOI:** 10.1038/s41598-018-21857-1

**Published:** 2018-02-28

**Authors:** M. Wierzba, M. Riegel, M. Wypych, K. Jednoróg, A. Grabowska, A. Marchewka

**Affiliations:** 1 Laboratory of Brain Imaging (LOBI), Neurobiology Center, Nencki Institute of Experimental Biology, Polish Academy of Sciences, Warsaw, Poland; 2 Laboratory of Psychophysiology, Department of Neurophysiology, Nencki Institute of Experimental Biology, Polish Academy of Sciences, Warsaw, Poland; 3 Faculty of Psychology, SWPS University of Social Sciences and Humanities, Warsaw, Poland

## Abstract

It is widely accepted that people differ in memory performance. The ability to control one’s memory depends on multiple factors, including the emotional properties of the memorized material. While it was widely demonstrated that emotion can facilitate memory, it is unclear how emotion modifies our ability to suppress memory. One of the reasons for the lack of consensus among researchers is that individual differences in memory performance were largely neglected in previous studies. We used the directed forgetting paradigm in an fMRI study, in which subjects viewed neutral and emotional words, which they were instructed to remember or to forget. Subsequently, subjects’ memory of these words was tested. Finally, they assessed the words on scales of valence, arousal, sadness and fear. We found that memory performance depended on instruction as reflected in the engagement of the lateral prefrontal cortex (lateral PFC), irrespective of emotional properties of words. While the lateral PFC engagement did not differ between neutral and emotional conditions, it correlated with behavioural performance when emotional – as opposed to neutral – words were presented. A deeper understanding of the underlying brain mechanisms is likely to require a study of individual differences in cognitive abilities to suppress memory.

## Introduction

## Mechanisms underlying cognitive control over memory

Memory can be shaped by various cognitive mechanisms, some of which happen involuntarily, while others are intentional. Traditional models of memory defined memory performance in terms of accuracy, capacity and durability. As a consequence, remembering was assumed to be deliberate and goal-directed, while forgetting – involuntary and incidental. Yet, these traditional models have recently been challenged by researchers who proposed that not only remembering, but also forgetting can be beneficial (see^1,2^ for a review). This called for a re-examination of existing models and opened up a new line of research, focused specifically on the mechanisms of cognitive control over memory. While theoretical models of memory typically distinguished between memory storage systems and executive control systems that support information processing (see^3^ for review), exerting control over memory requires these two systems to interact. New research paradigms designed to study the underlying mechanisms demonstrated that one can exert cognitive control over memory – not only to enhance it, but also to impair it^1,2^.

In fact, mechanisms of cognitive control over memory were most extensively discussed in relation to memory inhibition/suppression. It was noted that such cognitive control can be accomplished at different stages of memory (encoding or retrieval), as well as via different cognitive mechanisms^4^. For instance, at the encoding stage one can selectively become engaged in elaborative rehearsal of some items, while refraining from the rehearsal of others (*the selective rehearsal hypothesis*). Another possibility is that one makes a deliberate effort to disrupt the consolidation of a memory trace by inhibiting rehearsal (*the encoding suppression hypothesis*). At the retrieval stage, once a memory trace has been formed, one can try shifting the mental context or avoid cues that remind one of this trace (*the context shift hypothesis*). Finally, when exposed to a cue, one may try to suppress reflexive retrieval, making the remembered item less accessible (*the retrieval inhibition hypothesis*) (see^2^ for a review). Although several experimental paradigms have already been proposed to study memory suppression, each of these paradigms is able to capture only some of the postulated cognitive mechanisms.

For instance, encoding inhibition/suppression was investigated with the use of the *item-wise directed forgetting* paradigm^1,2,5,6^. During the *study part* (encoding) subjects are presented with items one by one, and after each item they are instructed to remember or to forget it. In the *test part* (retrieval) subjects indicate whether the presented items occurred previously, irrespective of the original instruction. Memory performance can be assessed separately for each instruction. Similarly, retrieval inhibition/suppression was studied with the use of the *list-wise directed forgetting* paradigm^1,2,5,6^, in which items are studied in series. After each series of items, one group of subjects is instructed to *remember* them, while another group is instructed (usually unexpectedly) to *forget* them. Another series of items is then presented, this time with the instruction to *remember* for both groups. Finally, memory performance is tested for both series in each group of subjects. Retrieval inhibition/suppression was also studied with the use of the *think/no-think* paradigm^1,2,7^. In this paradigm, participants begin with learning cue-target associations. In the subsequent *think/no-think* part, participants are required to recall a target item after each cue for the majority of trials, but for certain cues they are instructed to refrain from recalling a corresponding item. Finally, participants undergo the *test* part, in which they receive the studied cues and are asked to recall the corresponding target items.

## Brain regions recruited in cognitive control over memory

Early attempts to explain cognitive control over memory at the neuronal level^8–11^ emphasized the role of the lateral prefrontal cortex (lateral PFC). In general, it was observed that the left lateral PFC is engaged in attempts of encoding or retrieval, while the right lateral PFC is engaged in their inhibition/suppression. In particular, previous research revealed that the right inferior frontal gyrus (IFG), among other prefrontal regions, is particularly involved in behavioural inhibition^12,13^. On the other hand, right middle frontal gyrus (MidFG) was frequently suggested to play a key role in memory suppression^14^.

Also, it has been proposed that inhibitory control could be explained in terms of underlying interactions between brain regions^1,14–16^. In particular, increased activity of the right lateral PFC was related to inhibitory regulation of various processes, including: motor, memory, emotion^14^. Specifically, cognitive control was related to: regulation of the motor cortex via subthalamic nucleus, globus pallidus and thalamus in the case of motor control; of the hippocampus in the case of control over memory; of the amygdala in the case of emotion regulation^14^. Similarly, recent research demonstrated that right lateral PFC areas might regulate not only the activity of hippocampus, responsible for the conscious processing of a memory representation, but also of other regions, representing a more indirect expression of a memory representation. For instance, using visual stimuli (complex scenes and faces)^15^ showed that inhibition of a memory trace can be related to the regulation of regions involved in the reinstatement of sensory features of memory representations, e.g. visual cortex and fusiform face area (FFA).

While the majority of research on control over memory focused on the involvement of the lateral PFC, contribution of multiple, interacting regions is more likely to underlie the cognitive mechanisms behind inhibition^17–19^. Specifically^17^, proposed that inhibition is only one example of a whole class of cognitive control processes and that all these processes are supported by a network of brain regions, rather than the right IFG alone. In fact, the lateral PFC was demonstrated to belong to the frontoparietal control network^20^, also referred to as the central executive network, the cognitive control network or the multiple demand system^21–23^. The frontoparietal control network was shown to be extensively connected with other networks and to flexibly alter its interactions with other brain regions according to task demands^21^. Thus, it seems crucial to consider the contribution of other brain regions, especially when investigating complex interactions between various cognitive mechanisms.

## Role of emotion in cognitive control over memory

While it was widely demonstrated that emotion can facilitate our memory^24,25^, it is not well understood how emotion modifies our ability to inhibit or suppress memory^9,26–29^. Although emotional memories were demonstrated to be more persistent in comparison to neutral ones^9,27^, the underlying brain mechanisms are unclear. For instance^9^, reported that the encoding suppression of emotional (as compared to neutral) information was accompanied by more activity in the right lateral PFC. On the other hand^27^, found that encoding suppression yielded comparable activation of the right lateral PFC for both negative and neutral information. However, emotional (in contrast to neutral) items were reported to engage relatively less activity of the right PFC when suppression was successful^27^. Furthermore, as emotional valence and arousal were proposed to explain the effects of emotion on memory suppression^9,27^, it is also possible that these effects specifically relate to certain categories of emotional experience (e.g. fear, sadness, disgust)^26^. As pointed out by^28^, one of the main reasons for this apparent lack of consensus among researchers is that individuals differ in their cognitive performance. For instance, by accounting for individual differences in subjects’ performance^28^ were able to show that cognitive effort to suppress retrieval can result in reduced access to both mnemonic and emotional content. In particular, the better subjects were at suppressing retrieval of images, the more it reduced their emotional evaluation of these images. Moreover, they demonstrated that the right MidFG regulates the activity of hippocampus and amygdala in parallel during retrieval suppression of emotional information^28^. Thus, individual differences should be taken into account in order to fully understand brain mechanisms underlying memory suppression and how it is affected by emotion.

## Current study

In the current study, we examined cognitive control over memory, as well as the manner in which it is modulated by emotion, by taking individual differences into account. Since previous studies on the role of emotion in encoding suppression led to inconsistent conclusions, we decided to focus on the encoding process as well. We verified task-related brain activity patterns, as well as the underlying functional connectivity. Following previously reported fMRI results, we hypothesized that encoding attempt should engage the left, while encoding inhibition/suppression should engage the right lateral PFC areas. Furthermore, we specifically expected the right IFG and the right MidFG to be activated during encoding suppression. Given previously demonstrated functional connectivity results, we expected the activity of hippocampus to be modulated by the lateral PFC areas. Specifically, we hypothesized to find increased correlation between the activity of the hippocampus and the left lateral PFC during remembering. On the other hand, we expected decreased correlation between the activity in the hippocampus and the right lateral PFC during forgetting^14^. Following the findings of^15^, we decided to verify the inhibitory regulation over regions, which we expected to represent an indirect expression of memory representation in the case of verbal stimuli. Hence, during the suppression condition, we expected a decrease in correlation between activity in the right lateral PFC and activity in regions functionally related to verbal processing, i.e. visual word form area (VWFA)^30,31^. Finally, we expected to find different engagement of the lateral PFC in response to memory instructions for neutral and emotion trials^9,26,27^, as well as for trials representing different emotion categories^26^. Moreover, we anticipated that the engagement of the lateral PFC might be dependent on subjects’ memory performance. Similarly, we expected the engagement of the lateral PFC to be parametrically modulated by individual ratings of valence, arousal, sadness and fear.

## Materials and Methods

### Procedure

The experimental procedure was based on the item-wise *directed forgetting* paradigm and consisted of two parts: *study* and *test*. The procedure was implemented using Presentation (ver. 18.1 build 03.31.15; Neurobehavioral Systems, Inc., Albany, CA, USA). Behavioural data was obtained during the study and test parts. Simultaneous acquisition of MRI data was performed during the study part.

During the study part, subjects viewed 120 words (emotional or neutral), which they were instructed either to remember or to forget. Half of the words were followed by the instruction to remember (to-be-remembered, TBR), and half by the instruction to forget (to-be-forgotten, TBF). Each trial began with a fixation cross [0.5 s], followed by the presentation of a word [2 s], followed by another fixation cross [0.5 s] and the memory instruction [2 s]. Trials were separated from one another by intervals of random duration [7–9 s].

**Figure 1:**
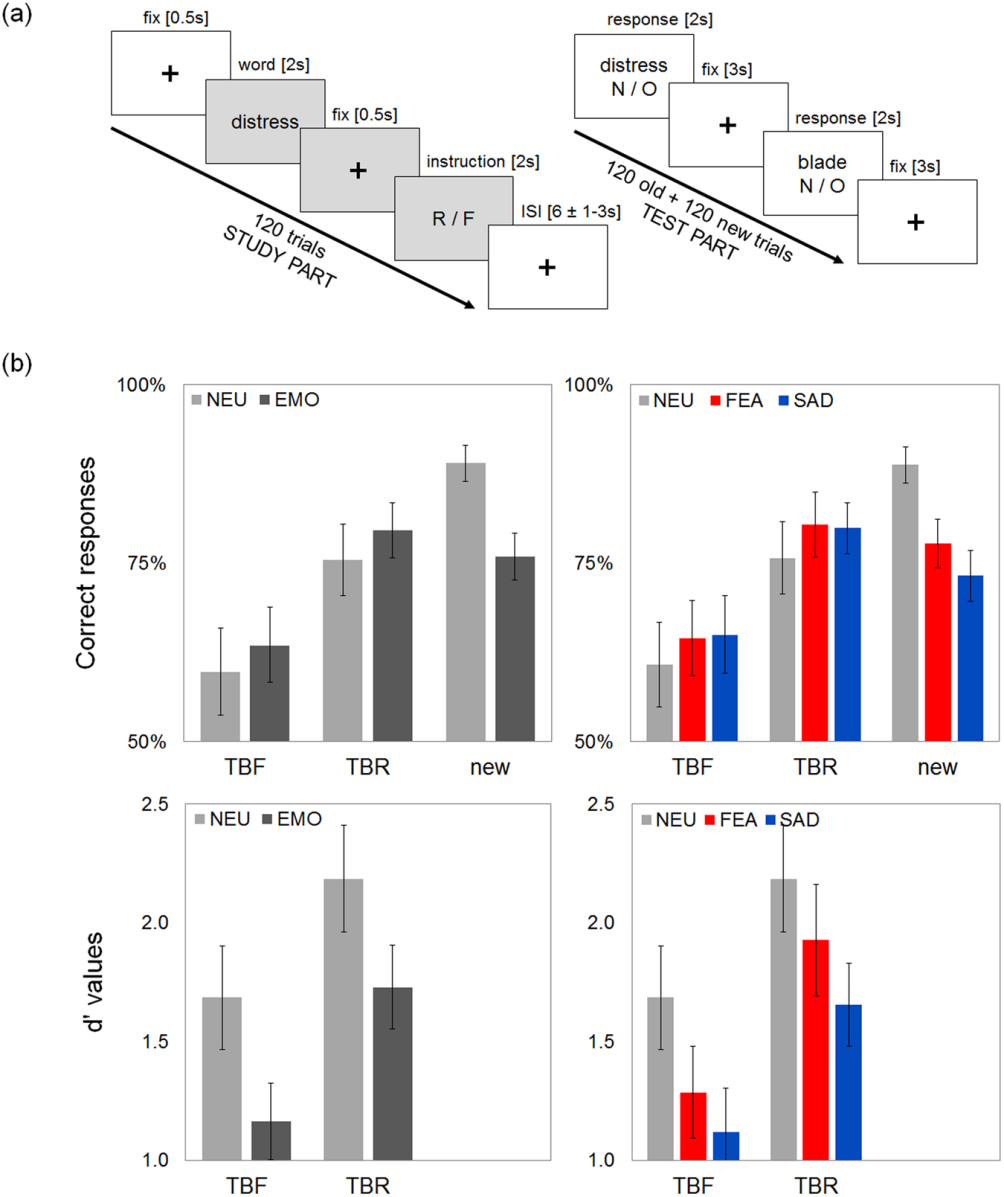
(**a**) A schematic representation of the experimental paradigm. (**b**) Memory performance (proportion of correct responses, as well as corresponding d’ values): for neutral (NEU) and emotional (EMO) words (left panel); for neutral (NEU), fear-related (FEA) and sadness-related (SAD) words (right panel). The directed forgetting effect was observed irrespective of emotional properties of the memorized material. Error bars represent the 95% confidence interval (CI) of a mean.

After a break of 30 minutes, the subjects participated in the test part, during which they viewed 120 old and 120 new words (emotional or neutral) and indicated whether the presented words had occurred previously, irrespective of the original instruction. Each trial consisted of word presentation [2 s], followed by a fixation cross [3 s]. The schematic illustration of the experimental design can be found in Fig. 1.

The order in which words were presented was randomized with the following constraints: no more than three trials in a row, assigned to the same type of experimental condition (TBR, TBF in case of study part; TBR, TBF, new in case of the test part) and no more than three trials in a row, representing the same emotion category.

On the following day, the subjects were required to complete the assessment of stimuli in terms of the elicited emotion (valence, arousal, sadness and fear).

The assessment task was preceded by brief instructions. Subjects were able to return to the instruction screen or ask for assistance when in doubt. The words were assessed one at a time. A single word was displayed along with the rating scales: valence, arousal, sadness and fear. The rating scales were identical to those described in^32^ and^33^. The valence scale ranged from -3 – *this word elicits very negative emotions in me*, to 3 - *this word elicits very positive emotions in me*. The arousal scale ranged from 1 – *I am not emotionally aroused*, to 5 - *I am emotionally aroused*. The sadness and fear scales ranged from: 1 – *this word does not elicit this emotion in me at all*/*only slightly elicits this emotion in me*, to 7 – *this word strongly elicits this emotion in me*/*elicits this emotion in me to a significant extent*. Subjects were encouraged to indicate their immediate, spontaneous reaction to words. As soon as a word was rated on each scale, the next screen with the subsequent word was displayed. No time constraints to complete the task were introduced.

### Materials

Stimuli were selected from the Nencki Affective Word List (NAWL^32,33^,) to represent three distinct emotion categories: neutral (NEU), sadness (SAD) and fear (FEA). The general selection rules were to minimize the variance of the emotion ratings within the category and to maximize the difference in the mean ratings between the categories. The neutral and emotion words were selected to differ with respect to valence and arousal. The sadness and fear words were selected to differ with respect to sadness and fear intensities, but were matched in terms of valence and arousal. Based on the above criteria, 120 neutral and 120 emotion (60 sadness, 60 fear) words were selected and assigned equally to respective experimental conditions. The assignment of the stimuli to emotion categories was further validated by individual ratings collected from the participants during the assessment task.

### Participants

As we had planned to use subjects’ individual ratings to explore emotion effects, we decided to limit the study sample to female volunteers to avoid the potentially confounding effect of gender. A total of 50 female subjects were invited to participate in the study. Several recruitment channels were used, including mailings, as well as social media. Most of the subjects were college students or young graduates from various faculties and departments of several universities and schools in Warsaw.

All 50 subjects (*M* = 25.0, *SD* = 2.1) completed the study and the test parts. Among those, 25 right-handed subjects (*M* = 24.6, *SD* = 2.3) were randomly chosen to undergo the simultaneous fMRI acquisition. A total of 46 subjects (*M* = 25.0, *SD* = 2.1) completed the assessment part, 24 of which (*M* = 24.6, *SD* = 2.4) were examined with MRI.

Subjects received financial gratification in the amount of PLN 50–100 (approximately EUR 10–20), depending on which sessions (behavioural, scanning, assessment) they completed. The Committee for Research Ethics of the Faculty of Psychology at the University of Warsaw (Komisja ds. Etyki Badań Naukowych, Wydział Psychologii, Uniwersytet Warszawski) approved the experimental protocol of the study. The experiment was carried out in accordance with American Psychological Association’s (APA) Ethical Principles of Psychologists and Code of Conduct (http://www.apa.org/ethics/code/). A written informed consent was obtained from each participant and the possibility to quit the experiment at any point without stating reasons was ensured.

### Behavioural data analysis

To assess the reliability of experimental manipulation, we compared the behavioural results from the *fMRI* and the *non-fMRI* samples by treating experiment as between-subjects factor. Thus, in the reported analyses we first establish the existence of the main effect of experiment (two levels: *non-fMRI* sample, *fMRI* sample), as well as of the interaction effect between experiment and other factors. We then discuss the effects of factors of interest collapsed across the experiments (*total* sample).

First, individual ratings data was used to validate the assignment of stimuli to respective emotion categories. Mean valence, arousal, sadness, and fear values were calculated for each word. Additionally, the mean valence, arousal, sadness, and fear ratings were calculated within emotion categories at the subject level. Then ANOVAs were performed with emotion category (three levels: NEU, SAD, FEA) as the within-subject factor and experiment as between-subjects factor, separately for each emotion parameter: valence, arousal, sadness, fear.

Next, task performance data was used to examine the general effects of memory control. Based on behavioural performance, trials were classified as either: hits (*old* as *old*), correct rejections (*new* as *new*), false alarms (*new* as *old*), or incorrect rejections (*old* as *new*). The corresponding proportion of correct responses (hits for TBR words, hits for TBF words, correct rejections for new words) were then calculated for each emotion category separately at the subject level. Subsequently, we considered subjects’ task performance with respect to signal detection theory. To this end, for each subject we computed d’ = Z(hits) − Z(false alarms), separately for each instruction and for each emotion category^34^. Similarly, we used difference in d’ values (d’ difference = d’TBR−d’TBF) to assess the magnitude of the directed forgetting effect for each subject, separately in each emotion category. Finally, ANOVAs were performed on these d’ values to test the effect of emotion on memory performance. In the first analysis, we defined the instruction (two levels: TBR, TBF) and emotion category (two levels: NEU, EMO) as within-subject factors, as well as experiment as between-subjects factor. In the second analysis, we explored more detailed effects by using the instruction (two levels: TBR, TBF) and emotion category (three levels: NEU, SAD, FEA) as within-subject factors, as well as experiment as between-subjects factor.

To further investigate the relationship between emotion and memory performance, correct and incorrect responses corresponding to each instruction (TBR, TBF, new) were characterized by computing mean valence, arousal, sadness and fear at the subject level based on individual ratings. ANOVAs were then performed, with memory outcome (two levels: correct, incorrect) and instruction (three levels: TBR, TBF, new) as within-subject factors, as well as experiment as between-subjects factor, separately for each emotion measure: valence, arousal, sadness, fear. Lastly, we explored the relationship between the emotional properties of a word (i.e. valence, arousal, sadness and fear) and the memorability of that word (i.e. proportion of subjects who correctly classified a given word as old or new). To this end, we performed pairwise correlation analyses separately for TBR, TBF, as well as new words.

### MRI data acquisition

Magnetic resonance imaging data was acquired using a 3T Siemens MAGNETOM Trio system (Siemens Medical Solutions) equipped with a 32-channel head coil. Within a single scanning session the following images where acquired: structural localizer image, first series of functional EPI images (TR: 2500 ms, TE: 27 ms, flip angle: 90°, voxel size: 3.5 × 3.5 × 3.5 mm, field of view: 224 mm, measurements: 314), second series of functional EPI images (same parameters), structural T1-weighted image (TR: 2530 ms, TE: 3.32 ms, flip angle: 7°, voxel size: 1 × 1 × 1 mm, field of view: 256 mm, measurements: 1), field map magnitude image (TR: 400 ms, TE: 6.81 ms, flip angle: 60°, voxel size: 3.5 × 3.5 × 3.5 mm, field of view: 224 mm, measurements: 2), field map phase image (TR: 400 ms, TE: 4.35 ms/6.81 ms, flip angle: 60°, voxel size: 3.5 × 3.5 × 3.5 mm, field of view: 224 mm, measurements: 1).

### MRI data analysis

#### Data preprocessing

DICOM series were converted to NIfTI with MRIConvert (ver. 2.0.7 build 369, https://lcni.uoregon.edu/downloads/mriconvert/mriconvert-and-mcverter). Spatial preprocessing was performed using Statistical Parametric Mapping (SPM12, http://www.fil.ion.ucl.ac.uk/spm/). Functional images were corrected for distortions related to magnetic field inhomogeneity; corrected for motion by realignment to the first acquired image; normalized to the MNI space and resliced to preserve the original resolution to 3.5 × 3.5 × 3.5 mm; and smoothed with the 6 mm FWHM Gaussian kernel. Prior to normalization, structural images were coregistered to the mean functional image; segmented into separate tissues using the default tissue probability maps; registered to the template generated from their own mean using the DARTEL approach; registered to the MNI space and resliced to preserve the original resolution to 1 × 1 × 1 mm.

For the purpose of functional connectivity analyses, functional images were further processed to identify the sources of artifacts using the Artifact Detection Toolbox (ART, http://www.nitrc.org/projects/artifact_detect/) included in CONN (ver. 17.c, https://www.nitrc.org/projects/conn/). *Liberal* settings were used (defined as the 99th percentile in normative sample).

#### ROI definitions

In the present work we used the Harvard-Oxford atlas (https://fsl.fmrib.ox.ac.uk/fsl/fslwiki/Atlases) to label the reported peaks of activations, as well as to define anatomical regions for the purpose of the ROI-based analyses. The Harvard-Oxford atlas provides sufficiently precise delineation of lateral prefrontal cortex, which was especially relevant to the present study goals. The anatomical ROI definitions used for the left lateral prefrontal cortex were as follows: SFG l (Superior Frontal Gyrus Left), MidFG l (Middle Frontal Gyrus Left), IFG tri l (Inferior Frontal Gyrus, pars triangularis Left), IFG oper l (Inferior Frontal Gyrus, pars opercularis Left), FOrb l (Frontal Orbital Cortex Left), FP l (Frontal Pole Left). The anatomical ROI definitions used for the right lateral prefrontal cortex were as follows: SFG r (Superior Frontal Gyrus Right), MidFG r (Middle Frontal Gyrus Right), IFG tri r (Inferior Frontal Gyrus, pars triangularis Right), IFG oper r (Inferior Frontal Gyrus, pars opercularis Right), FOrb r (Frontal Orbital Cortex Right), FP r (Frontal Pole Right). Also, another goal of the present study was to test for the interactions between the lateral prefrontal cortex and regions hypothesized to participate in the memory formation process: hippocampus, as well as visual word form area (VWFA), The ROI definitions used for the hippocampus were: Hippocampus r, Hippocampus l. In the case of the visual word form area (VWFA) we decided to use a coordinate-based spherical mask (with a radius of 10 mm), as it is rather difficult to delineate the VWFA based on anatomical landmarks. Following the reviews by^30,31^, the center of the visual word form area was located at approximately x = −43, y = −54, z = −12 as defined in Talairach space, which corresponds to x = −44, y = −54, z = −18 as defined in MNI space (mm, to the left, posterior, and below the anterior commissure, respectively).

#### Functional anatomical mapping analysis

Subject-level and group-level analyses were performed using the mass-univariate approach, based on the general linear model, as implemented in SPM12. Additional ROI analysis was performed using the MarsBaR (http://marsbar.sourceforge.net/index.html) toolbox.

At the subject level, each single event was modeled with onset corresponding to the presentation of a word and duration of 4.5 seconds (representing both word and memory instruction processing). A default canonical hemodynamic response function (HRF) with no derivatives was used to approximate the expected BOLD signal. Motion parameters (translation in x, y, z directions; rotation around x, y, z axes) were inserted into each model as covariates, resulting in 6 regressors of no interest per session. A default high-pass filter cutoff of 128 seconds was used to remove low-frequency signal drifts.

Next, at the subject level functional volumes were split into conditions depending on instruction (TBR, TBF) and emotion category (emotional, neutral). As a result, the following conditions were specified: NEU TBR, EMO TBR, NEU TBF, EMO TBF. The number of trials falling into each condition was identical for each subject. At the group level, we performed ANOVA in flexible factorial design with instruction (two levels: TBR, TBF) and emotion (two levels: EMO, NEU) as within-subject factors, as well as the subject factor. The interaction effect between instruction and emotion was included to the design matrix. Next, the following contrasts were tested in both directions in the whole brain analyses: instruction effect among *all trials*: (NEU TBR & EMO TBR) – (NEU TBF & EMO TBF), instruction effect among *NEU trials*: NEU TBR – NEU TBF, as well as among *EMO trials*: EMO TBR – EMO TBF. Additionally, paired t-tests were performed for direct comparison between (NEU TBR – NEU TBF) and (EMO TBR – EMO TBF) contrasts. A voxel-wise height threshold of *p* < 0.001 (uncorrected) combined with a cluster-level extent threshold of *p* < 0.05 (corrected for multiple comparisons using the FWE rate) was applied in the whole brain analyses.

To further explore any potential difference in instruction effects between emotional and neutral words, contrast estimate values extracted from the paired t-test analysis were used in subsequent ROI analysis. For encoding attempt ((NEU TBR > NEU TBF) – (EMO TBR > EMO TBF)) these were left lateral prefrontal gyri (see *ROI definitions* section), whereas for encoding suppression ((NEU TBF > NEU TBR) – (EMO TBF > EMO TBR)) these were right lateral prefrontal gyri (see *ROI definitions* section).

The coordinates of significant effects are reported in MNI space and were labeled according to Harvard-Oxford atlas with the use of bspmview (http://www.bobspunt.com/bspmview). Results were visualized with the use of BrainNet Viewer (https://www.nitrc.org/projects/bnv).

#### Parametric modulations analysis

In the parametric modulations analysis we investigated whether variable emotional features of the stimuli can be reflected in brain activity. At the subject level, additional regressors modulated by the individual ratings of valence, arousal, sadness or fear were modeled. Thus, for each initial regressor a parametrically modulated regressor was created. Each subject’s individual ratings were included in the respective experimental conditions as linear (first order) modulators. At this point, the variance of a parameter vector was assessed in each condition to discard conditions with zero variance from further analysis. A single parameter at a time was added to the subject-level model, to avoid orthogonalization effects. At the group level, we used the parametrically modulated regressors to perform ANOVA for each parameter separately, by defining a within-subject factor representing instruction, as well as the subject factor. Subsequently, we tested the effect of the instruction to remember (TBR > baseline) and the effect of the instruction to forget (TBF > baseline) as correlated with a given parameter. Depending on the values a given parameter held, we identified regions whose activity was correlated positively or negatively with the parameter. Specifically, we looked at the negative correlation with valence (i.e. the lower the VAL score, the more emotional the given word), and at the positive correlation with arousal, sadness and fear (i.e. the higher the ARO/SAD/FEA score, the more emotional the given word).

#### Functional connectivity analysis

The CONN (ver. 17.c, https://www.nitrc.org/projects/conn) software was used to perform task-related functional connectivity analysis. Subject-level SPM models specified in terms of instruction (TBR, TBF) and emotion category (emotional, neutral) were used to analyze task-related connectivity changes.

Condition definitions along with the corresponding onsets and durations of the events were imported from SPM models. Structural and functional images already preprocessed in SPM12, i.e. images aligned to the MNI template, were used. Voxel-level BOLD timeseries were extracted from spatially smoothed functional volumes, while ROI-level BOLD timeseries were extracted from the corresponding unsmoothed functional volumes.

To remove further confounding physiological and motion artifactual effects from the BOLD timeseries before computing connectivity measures, the denoising procedure implemented in CONN was performed. For that purpose, signal from white matter and cerebrospinal fluid masks, as well as subject-level motion-related covariates (realignment parameters previously obtained with the SPM preprocessing, as well as motion outliers identified with ART) were regressed out from the signal. Furthermore, the main condition effects (condition events convolved with HRF) were included as confounds to avoid connectivity between two areas driven by shared task-related responses, but to focus on the remaining functional connectivity effects. The signal was high-pass filtered in the range above 0.008 Hz to keep higher-frequency information related to the task.

The generalized psychophysiological interaction (gPPI) analysis was used to model subject-level functional connectivity. The ROI-level BOLD timeseries were used to compute ROI-to-ROI correlations at the group level. Again, instruction effects were tested for all trials: (NEU TBR & EMO TBR) – (NEU TBF & EMO TBF), for NEU trials: NEU TBR – NEU TBF, as well as for EMO trials: EMO TBR – EMO TBF. To test the effects of encoding attempt (TBR > TBF), ROI-to-ROI correlations were computed among the left lateral prefrontal ROIs and the hippocampi, as well as among the left lateral prefrontal ROIs and the VWFA. Similarly, to test the effects of encoding suppression (TBF > TBR), ROI-to-ROI correlations were computed among the right lateral prefrontal ROIs and the hippocampi, as well as among the right lateral prefrontal ROIs and the VWFA. Detailed description of the ROIs can be found in the *ROI definitions* section. A two-sided threshold of *p* < 0.05 (corrected for multiple comparisons using the FDR rate at the seed-level) was applied to report both increases and decreases in functional connectivity.

## Results

### Behavioural results

#### Individual ratings

**Figure 2:**
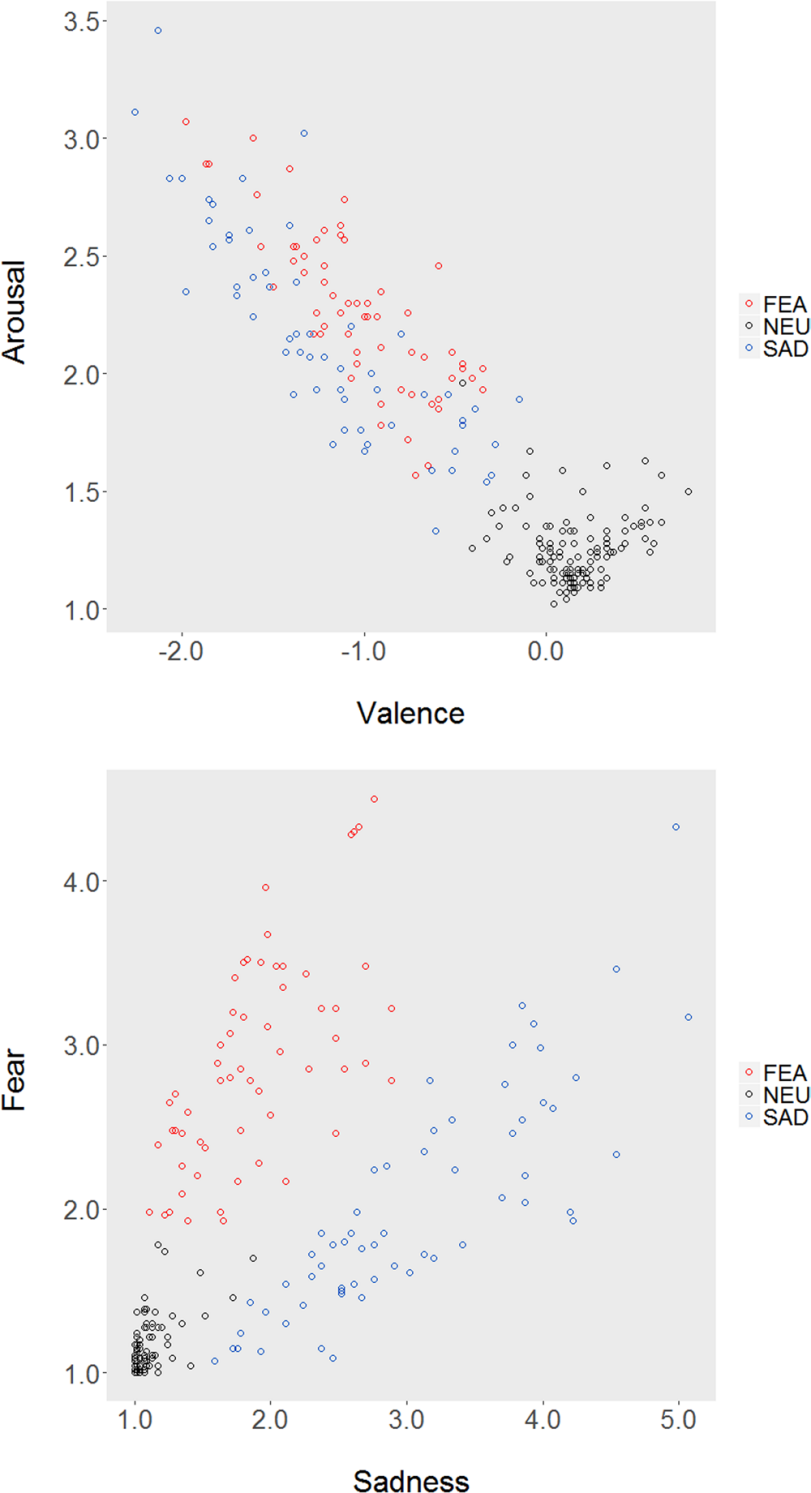
Distribution of mean valence, arousal, sadness and fear ratings for each word. Colors represent stimulus categories: FEA – fear, NEU – neutral, SAD – sadness. Included are subjects from both *non-fMRI* and *fMRI* samples, who completed the assessment part (*n* = 46).

Mean valence, arousal, sadness and fear ratings from the *total* sample, calculated for each word separately, are plotted in Fig. 2 and the corresponding pairwise correlation coefficients are summarized in Table 1.

**Table 1:** Pairwise correlation coefficients for valence, arousal, sadness and fear ratings for each word. Included are subjects from both *non-fMRI* and *fMRI* samples, who completed the assessment part (*n* = 46) Significant correlations are marked with an asterisk (p < 0.01, two-sided).

	Pearson’s correlation			
	VAL	ARO	SAD	FEA
VAL	1			
ARO	−0.90*	1		
SAD	−0.88*	0.81*	1	
FEA	−0.84*	0.92*	0.61*	1

The analysis performed on the mean ratings in each emotion category revealed no effect of experiment (valence, F(1, 44) = 1.04, *p* = 0.31, *η*^2^ = 0.02; arousal, F(1, 44) = 0.54, *p* = 0.47, *η*^2^ = 0.01; sadness, F(1, 44) = 2.59, *p* = 0.11, *η*^2^ = 0.05; fear, F(1, 44) = 3.77, *p* = 0.06, *η*^2^ = 0.08), nor interaction between experiment and emotion category (valence, F(1.51, 66.48) = 0.89, *p* = 0.39, *η*^2^ = 0.02; arousal, F(1.81, 79.83) = 0.63, *p* = 0.52, *η*^2^ = 0.01; sadness, F(1.61, 70.78) = 1.34, *p* = 0.26, *η*^2^ = 0.03; fear, F(1.50, 65.91) = 1.89, *p* = 0.17, *η*^2^ = 0.04), in each of the tested parameters. Finally, a significant main effect of emotion category was found (valence, F(1.51, 66.48) = 199.18, *p* < 0.05, *η*^2^ = 0.82; arousal, F(1.81, 79.83) = 106.53, *p* < 0.05, *η*^2^ = 0.71; sadness, F(1.61, 70.78) = 143.31, *p* < 0.05, *η*^2^ = 0.76; fear, F(1.50, 65.91) = 101.33, *p* < 0.05, *η*^2^ = 0.70) for all tested parameters.

Further Bonferroni corrected post hoc tests resulted in a significant difference in valence (*p* < 0.05), but no difference in arousal (*p* = 0.20) between SAD and FEA words. Yet, both categories were rated as having a significantly stronger emotional load than words in the NEU category (*p* < 0.05, in each pairwise comparison). Moreover, NEU, SAD and FEA categories differed from each other in terms of sadness and fear (p < 0.05, in each pairwise comparison).The detailed results are presented in Table 2.

**Table 2:** Estimated marginal means of valence, arousal, sadness and fear ratings calculated for neutral (NEU), fear (FEA) and sadness (SAD) stimulus categories. Results for *non-fMRI* (*n* = 22) and *fMRI* (*n* = 24) samples, as well as for the *total* sample (*n* = 46).

								95% Confidence Interval					
		Mean			Std. Error			Low.	Upp.	Low.	Upp.	Low.	Upp.
		non-fMRI	fMRI	total	non-fMRI	fMRI	total	non-fMRI		fMRI		total	
valence	NEU	0.15	0.17	0.16	0.04	0.04	0.03	0.07	0.24	0.09	0.26	0.10	0.23
	FEA	−0.96	−1.10	−1.03	0.12	0.12	0.08	−1.20	−0.72	−1.33	−0.87	−1.20	−0.87
	SAD	−1.12	−1.28	−1.20	0.09	0.09	0.07	−1.31	−0.93	−1.46	−1.10	−1.33	−1.07
arousal	NEU	1.24	1.27	1.25	0.05	0.05	0.03	1.14	1.34	1.17	1.36	1.18	1.32
	FEA	2.24	2.30	2.27	0.14	0.13	0.10	1.96	2.52	2.03	2.57	2.07	2.46
	SAD	2.04	2.23	2.14	0.12	0.12	0.08	1.80	2.29	2.00	2.46	1.97	2.31
sadness	NEU	1.06	1.10	1.08	0.02	0.02	0.01	1.02	1.10	1.06	1.13	1.05	1.10
	FEA	1.68	2.08	1.88	0.15	0.14	0.10	1.38	1.97	1.80	2.36	1.68	2.08
	SAD	2.88	3.20	3.04	0.21	0.20	0.15	2.45	3.31	2.79	3.61	2.74	3.34
fear	NEU	1.09	1.16	1.12	0.04	0.04	0.03	1.01	1.16	1.09	1.23	1.07	1.17
	FEA	2.65	3.12	2.88	0.24	0.23	0.17	2.16	3.13	2.65	3.58	2.55	3.22
	SAD	1.75	2.26	2.00	0.15	0.15	0.11	1.44	2.06	1.96	2.55	1.79	2.22

#### Memory performance

Task performance as expressed in the proportion of correct responses (hits for TBR words, hits for TBF words, correct rejections for new words), as well as corresponding d’ values are summarized in Fig. 1. These summary statistics demonstrate instruction impact on memory performance referred to as the directed forgetting effect: better memory performance for TBR words, than for TBF words. Specifically, hit rate was higher for TBR words, than for TBF words, and the difference between the two hit rates was weaker for emotional as compared to neutral words. Nevertheless the observed difference owing to emotion was rather weak and was even more negligible between emotion categories (NEU, FEA, SAD). On the other hand, emotional words produced more false alarms in response to new words. To be able to account for both changes: in hits, as well as in false alarms, we used d’ values instead of proportions of correct responses in the subsequent analyses.

In the first analysis, we used the instruction (two levels: TBR, TBF) and emotion category (two levels: NEU, EMO) as within-subject factors, as well as experiment as between-subjects factor. No effect of experiment was found (F(1, 48) = 0.97, *p* = 0.33, *η*^2^ = 0.02). Also, there was no interaction between the experiment and emotion category factors (F(1, 48) = 0.08, *p* = 0.78, *η*^2^ = 0), that is emotion category had comparable effect on memory performance in both samples. Interestingly, a significant interaction was found between the experiment and instruction factors (F(1, 48) = 6.39, *p* < 0.05, *η*^2^ = 0.12), with the instruction effect being stronger in *non-fMRI* sample, as compared to the *fMRI* sample. Finally, no interaction was found between the experiment, emotion category and instruction factors (F(1, 48) = 0.38, *p* = 0.54, *η*^2^ = 0). Overall, the analysis revealed a significant main effect of instruction (F(1, 48) = 59.74, *p* < 0.05, *η*^2^ = 0.55). Specifically, higher d’ for to-be-remembered (TBR) than for to-be-forgotten (TBF) items was found. This effect was present irrespective of emotion category, demonstrated by the lack of interaction effect between emotion category and instruction (F(1, 48) = 0.53, *p* = 0.47, *η*^2^ = 0.01).

In the second analysis, we used the instruction (two levels: TBR, TBF) and emotion (three levels: NEU, SAD, FEA) as within-subject factors, as well as experiment as between-subjects factor. Again, no effect of experiment was found (F(1, 48) = 1.06, *p* = 0.31, *η*^2^ = 0.02). There was no interaction between the experiment and emotion category factors (F(1.99, 95.48) = 0.25, *p* = 0.78, *η*^2^ = 0), while a significant interaction between the experiment and instruction factors (F(1, 48) = 5.82, *p* < 0.05, *η*^2^ = 0.11) with the instruction effect being stronger in *non-fMRI* sample, as compared to the *fMRI* sample. Similarly, no interaction was found between the experiment, emotion category and instruction factors (F(1.99, 95.74) = 2.59, *p* = 0.08, *η*^2^ = 0.05). Overall, a significant main effect of instruction was found (F(1, 48) = 63.17, *p* < 0.05, *η*^2^ = 0.57) with higher d’ for to-be-remembered (TBR) than for to-be-forgotten (TBF) items. Again, this effect was present irrespective of emotion category, as revealed by the lack of interaction effect between emotion category and instruction (F(1.99, 95.74) = 0.98, *p* = 0.38, *η*^2^ = 0.02).

#### Individual ratings × memory performance

The analysis of the relation between individual ratings and task performance revealed no effect of experiment (valence, F(1, 44) = 0.37, *p* = 0.55, *η*^2^ = 0; arousal, F(1, 44) = 0.58, *p* = 0.45, *η*^2^ = 0.01; sadness, F(1, 44) = 3.11, *p* = 0.08, *η*^2^ = 0.07; fear, F(1, 44) = 3.59, *p* = 0.06, *η*^2^ = 0.08). Also, there was no interaction between the experiment and instruction (valence, F(1.95, 85.88) = 0.87, *p* = 0.42, *η*^2^ = 0.02; arousal, F(1.81, 79.82) = 2.22, *p* = 0.12, *η*^2^ = 0.05; sadness, F(1.83, 80.55) = 1.63, *p* = 0.20, *η*^2^ = 0.04; fear, F(1.73, 76.00) = 0.32, *p* = 0.69, *η*^2^ = 0), between experiment and memory outcome (valence, F(1, 44) = 0.24, *p* = 0.62, *η*^2^ = 0; arousal, F(1, 44) = 0.24, *p* = 0.62, *η*^2^ = 0; sadness, F(1, 44) = 1.68, *p* = 0.20, *η*^2^ = 0.04; fear, F(1, 44) = 0.02, *p* = 0.88, *η*^2^ = 0), nor between experiment, instruction and memory outcome considered together (valence, F(1.54, 67.78) = 1.19, *p* = 0.30, *η*^2^ = 0.03; arousal, F(1.74, 76.39) = 0.56, *p* = 0.55, *η*^2^ = 0.01; sadness, F(1.51, 66.30) = 1.30, *p* = 0.27, *η*^2^ = 0.03; fear, F(1.55, 68.05) = 0.37, *p* = 0.64, *η*^2^ = 0).

Overall, the analysis revealed a significant main effect of instruction (valence, F(1.95, 85.88) = 18.01, *p* < 0.05, *η*^2^ = 0.29; arousal, F(1.81, 79.82) = 14.14, *p* < 0.05, *η*^2^ = 0.24; sadness, F(1.83, 80.55) = 19.39, *p* < 0.05, *η*^2^ = 0.31; fear, F(1.73, 76) = 17.31, *p* < 0.05, *η*^2^ = 0.28), as well as interaction between instruction and memory outcome (valence, F(1.54, 67.78) = 29.08, *p* < 0.05, *η*^2^ = 0.40; arousal, F(1.74, 76.39) = 33.58, *p* < 0.05, *η*^2^ = 0.43; sadness, F(1.51, 66.30) = 25.18, *p* < 0.05, *η*^2^ = 0.36; fear, F(1.55, 68.05) = 24.47, *p* < 0.05, *η*^2^ = 0.36).

**Figure 3:**
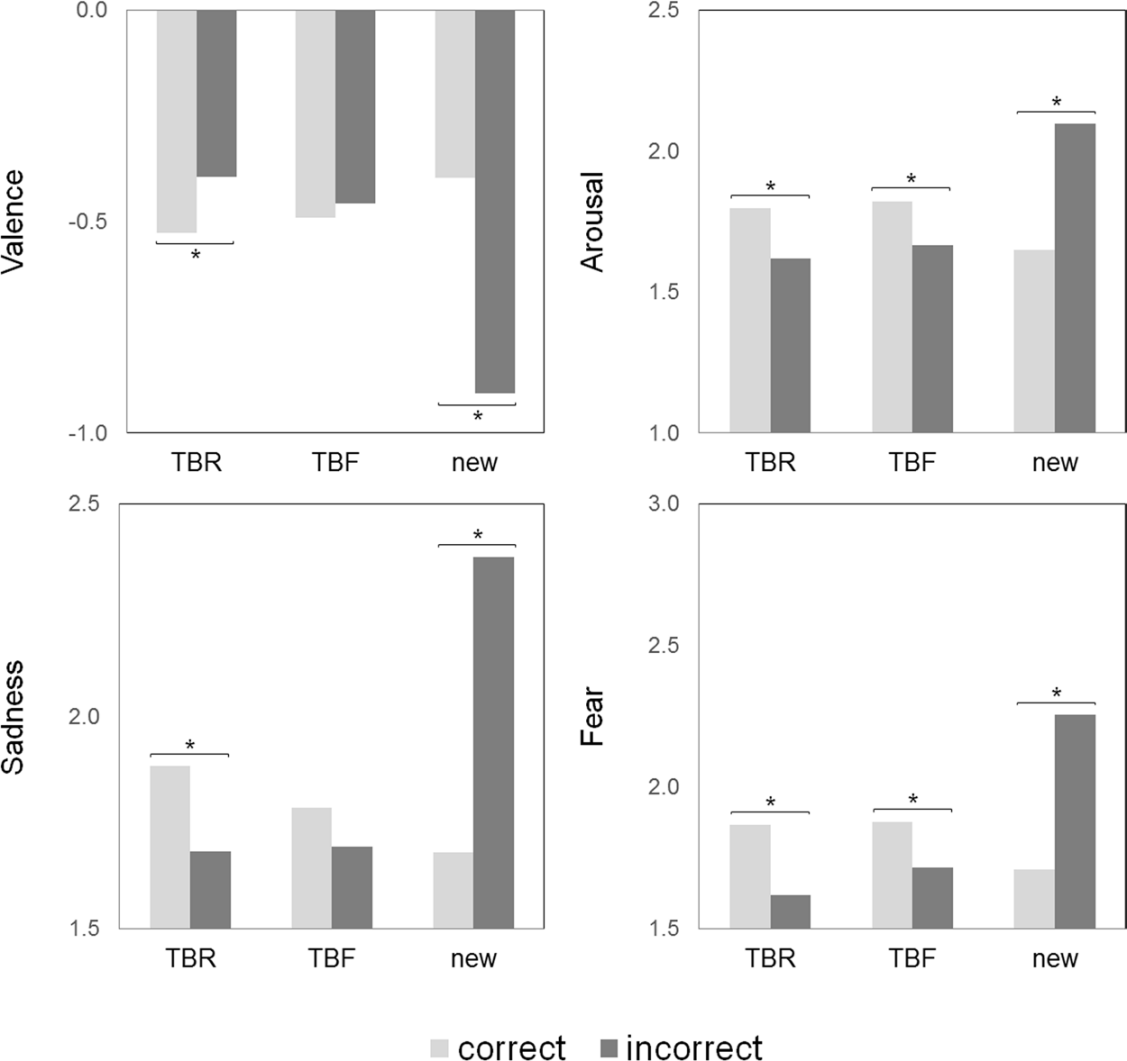
Mean valence, arousal, sadness and fear ratings calculated separately for correct and incorrect: TBR (to-be-remembered), TBF (to-be-forgotten), as well as new items. Depending on the trial type emotion influenced task performance in opposite ways: improved performance for studied, old items (higher scores of VAL, ARO, SAD, FEA for words in the set of correct recognitions) and impaired performance for unstudied, new items (higher scores of VAL, ARO, SAD, FEA for words in the set of false alarms). Significant differences are marked with an asterisk (*p* < 0.05).

Further Bonferroni corrected post hoc tests across all tested emotion measures revealed that, among TBR words, correct trials (as compared to incorrect trials) were assessed as having a stronger emotional load in terms of valence, arousal, sadness, as well as fear (*p* < 0.05, in each pairwise comparison); while among the new words incorrect trials (as compared to correct trials) were found to have a stronger emotional load (*p* < 0.05, in each pairwise comparison). The results were not consistent among TBF words, yet they remained similar to that observed among the TBR words. The summary of these results can be found in Fig. 3.

Finally, we used individual ratings and subjects’ task performance to index all words in terms of their emotional properties (i.e. valence, arousal, sadness and fear), as well as their memorability (i.e. proportion of subjects who correctly recognized a given word as old or new). The pairwise correlation analysis performed separately for TBR, TBF and new words revealed that memorability of a word was accompanied by its emotional properties only in the case of new words (*p* < 0.01, for each pairwise correlation coefficient), and not in the case of TBR and TBF words. Detailed results are summarized in Table 3.

**Table 3:** Pairwise correlation coefficients demonstrating the relationship between memorability and emotional properties of words. Memorability of each word was expressed as the proportion of subjects who correctly recognised a given word as old or new. Emotional properties of each word were expressed in terms of its mean valence, arousal, sadness and fear, based on ratings of subjects who completed the assessment part (*n* = 46). Significant correlations are marked with an asterisk (p < 0.01, two-sided).

	Pearson’s correlation			
	VAL	ARO	SAD	FEA
TBF (*n* = 60)	−0.14	0.16	0.10	0.12
TBR (*n* = 60)	−0.18	0.22	0.17	0.19
new (*n* = 120)	0.48*	−0.46*	−0.53*	−0.34*

### Functional anatomical mapping results

**Figure 4:**
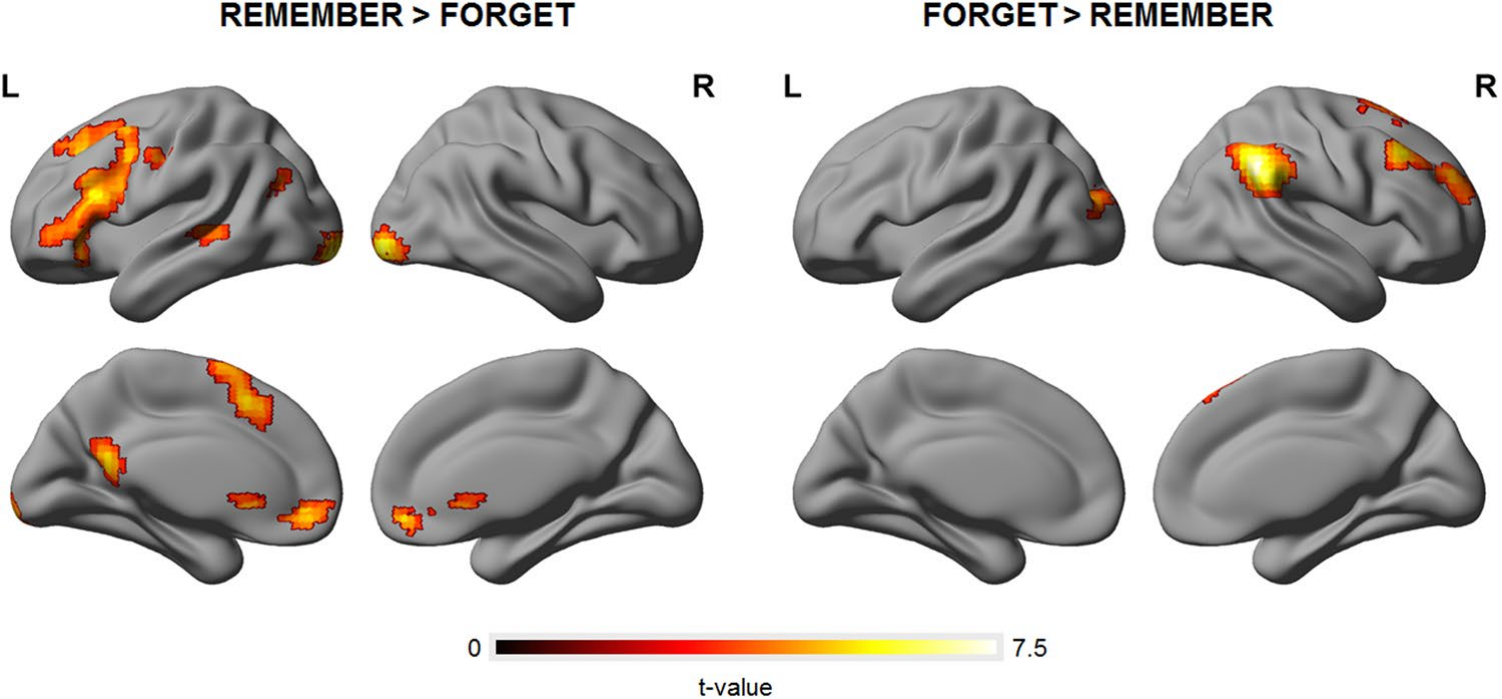
Whole-brain statistical parametric maps representing brain activation specific to encoding (TBR > TBF) and encoding suppression (TBF > TBR). The left lateral PFC were specifically involved in encoding, whereas the right lateral PFC in the suppression of encoding. A voxel-wise height threshold of *p* < 0.001 (uncorrected) combined with a cluster-level extent threshold of *p* < 0.05 (corrected for multiple comparisons using the FWE rate) was applied.

**Figure 5:**
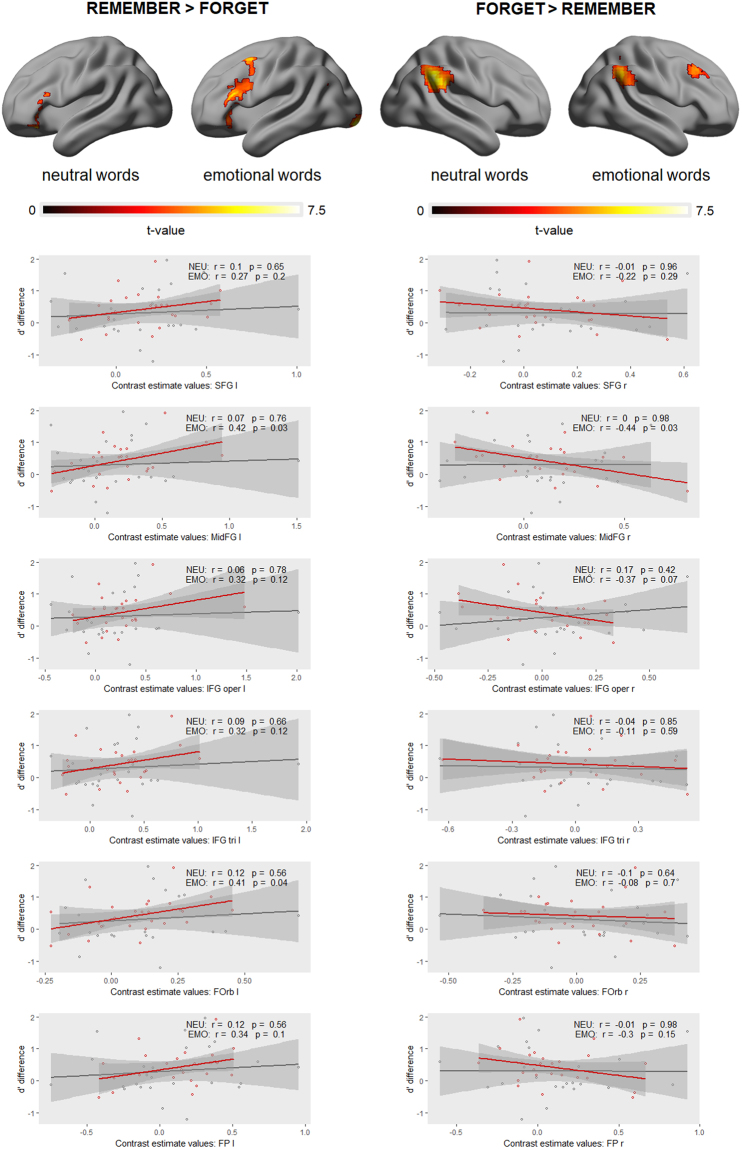
Whole-brain statistical parametric maps representing brain activation specific to encoding (TBR > TBF), as well as encoding suppression (TBF > TBR) during emotional (EMO) and neutral (NEU) conditions. A voxel-wise height threshold of *p* < 0.001 (uncorrected) combined with a cluster-level extent threshold of *p* < 0.05 (corrected for multiple comparisons using the FWE rate) was applied. Each activation map is accompanied by a plots demonstrating the relationship between brain activation and behavioural performance in EMO (red colour) and NEU (grey colour). The strength of the lateral PFC activation depended on subjects’ cognitive abilities to control their memory, specifically in the case of emotional as compared to neutral items. The strength of the activation was expressed in the contrast estimate values representing respective instruction effects extracted for a given ROI individually for each subject. In the case of encoding, contrast estimate values were extracted for anatomical masks representing the left lateral prefrontal cortex, whereas in the case of encoding suppression – the right lateral prefrontal cortex. Behavioural performance was expressed in the magnitude of the directed forgetting effect (difference in d’ values = d’TBR−d’TBF) estimated for each subject.

The whole-brain analyses revealed that the encoding attempt engaged left, while the encoding suppression engaged right lateral prefrontal regions (Fig. 4, Table 4). In particular, instruction to remember (TBR > TBF) activated the triangular part of the left IFG, as well as the left MidFG and SFG. On the other hand, instruction to forget (TBF > TBR) was found to be reflected in the activity of the right MidFG and the right SFG. These lateral prefrontal activations corresponding to the instruction effects appeared to be stronger in the case emotional as compared to neutral words (Fig. 5, Table 4). Nevertheless, whole-brain paired t-test comparisons between emotional and neutral words revealed no significant difference in the effect of instruction to remember (EMO TBR > EMO TBF) – (NEU TBR > NEU TBF), nor in the effect of instruction to forget (EMO TBF > EMO TBR) – (NEU TBF > NEU TBR).

**Table 4:** Peak level activations related to cognitive control over memory: encoding (TBR > TBF) and encoding suppression (TBF > TBR). Reported are comparisons done among all trials, as well as among neutral (NEU) and emotional (EMO) trials. Notation: TBR – to-be-remembered items, TBF – to-be-forgotten items. A voxel-wise height threshold of *p* < 0.001 (uncorrected) combined with a cluster-level extent threshold of *p* < 0.05 (corrected for multiple comparisons using the FWE rate) was applied. Activations surviving a peak-level FWE-corrected threshold of p < 0.05 are marked with an asterisk. Table shows all local maxima separated by more than 8 mm. Regions were automatically labelled using the Harvard-Oxford atlas. x, y, and z Montreal Neurological Institute (MNI) coordinates in the left-right, anterior-posterior, and inferior-superior dimensions, respectively.

Harvard-Oxford label of the peak	Cluster extent	Hemisphere	t-value	MNI coordinates			*p*_FWE_
				x	y	z	
**TBR > TBF**							
Occipital Pole	91	L	7.2527	−28	−91	−7	**0**.000*
Occipital Pole	76	R	6.597	28	−94.5	−7	**0**.000*
Inferior Frontal Gyrus pars triangularis	860	L	6.0089	−49	24.5	21	**0**.001*
Superior Frontal Gyrus		L	5.2155	−7	17.5	49	**0**.023*
Middle Frontal Gyrus		L	5.1828	−31.5	10.5	56	**0**.025*
Left Caudate	283	L	5.5811	−7	17.5	0	**0**.007*
Frontal Medial Cortex		R	5.3484	7	49	−10.5	**0**.015*
Right Caudate		R	5.308	7	17.5	0	**0**.017*
Cingulate Gyrus posterior division	80	L	5.2425	−3.5	−52.5	14	**0**.021*
*location not in atlas*	79	L	4.4351	−35	−63	21	0.242
Lateral Occipital Cortex superior division		L	4.1328	−45.5	−70	28	0.489
Lateral Occipital Cortex superior division		L	3.884	−31.5	−73.5	38.5	0.736
Superior Temporal Gyrus posterior division	78	L	4.2754	−59.5	−31.5	3.5	0.359
Middle Temporal Gyrus posterior division		L	4.1394	−56	−38.5	0	0.482
**TBF > TBR**							
Angular Gyrus	327	R	7.246	56	−49	35	**0**.000*
Angular Gyrus		R	4.4547	42	−52.5	52.5	0.230
Superior Frontal Gyrus	61	R	5.5532	14	17.5	59.5	**0**.007*
Superior Frontal Gyrus		R	3.7529	10.5	35	52.5	0.847
Middle Frontal Gyrus	191	R	5.5263	38.5	24.5	42	**0**.008*
Frontal Pole		R	5.0255	21	49	28	**0**.042*
Middle Frontal Gyrus		R	4.1272	35	35	38.5	0.494
Lateral Occipital Cortex superior division	88	L	4.8141	−28	−84	17.5	**0**.082*
**NEU: TBR > TBF**							
Left Accumbens	89	L	5.460	−7	14	−4	**0**.010*
Right Caudate		R	4.353	7	18	0	0.298
Occipital Pole	57	L	5.381	−28	−91	−7	**0**.013*
Middle Frontal Gyrus	175	L	4.782	−53	28	28	0.091
Inferior Frontal Gyrus, pars triangularis		L	4.532	−49	25	21	0.187
Superior Frontal Gyrus	153	L	4.648	−21	25	42	0.135
Superior Frontal Gyrus		L	4.556	−7	18	49	0.175
Juxtapositional Lobule Cortex (formerly Supplementary Motor Cortex)		L	3.789	−4	7	63	0.820
Frontal Medial Cortex	65	L	4.380	−11	49	−11	0.279
Frontal Medial Cortex		R	3.917	7	49	−11	0.704
**EMO: TBR > TBF**							
Occipital Pole	77	L	6.085	−25	−95	−11	**0**.000*
Occipital Pole		R	6.067	28	−95	−7	**0**.001*
Inferior Frontal Gyrus, pars opercularis	305	L	4.988	−49	21	18	**0**.048*
Superior Frontal Gyrus	53	L	4.320	−4	11	67	0.323
Superior Frontal Gyrus		L	3.579	−7	18	49	0.946
**NEU: TBF > TBR**							
Angular Gyrus	257	R	6.446	56	−49	32	**0**.000*
**EMO: TBF > TBR**							
Angular Gyrus	170	R	5.098	53	−49	39	**0**.033*
Angular Gyrus		R	4.202	42	−53	53	0.424
Middle Frontal Gyrus	63	R	4.537	42	25	46	0.185
Middle Frontal Gyrus		R	3.559	39	35	39	0.953

The abovementioned comparisons were further explored in a subsequent ROI analysis, in which contrast estimate values were extracted for each ROI defined within the lateral prefrontal cortex (see *ROI definitions* section). Again, the comparisons of the contrast estimates representing instruction effects (instruction to remember, instruction to forget) between emotional and neutral words revealed no significant differences. However, correlation analysis of these contrast estimate values and the magnitude of the directed forgetting effect (difference in d’ values = d’TBR−d’TBF) showed a significant relationship specifically for emotional words, and not for neutral words. In the case of encoding attempt (TBR > TBF), the correspondence to behavioural performance was especially strong for the orbital part of the left IFG, as well as left MidFG. In the case of encoding suppression (TBF > TBR), the right MidFG activity was shown to be the most strongly related to task performance. The corresponding correlation plots can be found in Fig. 5.

### Parametric modulations results

**Figure 6:**
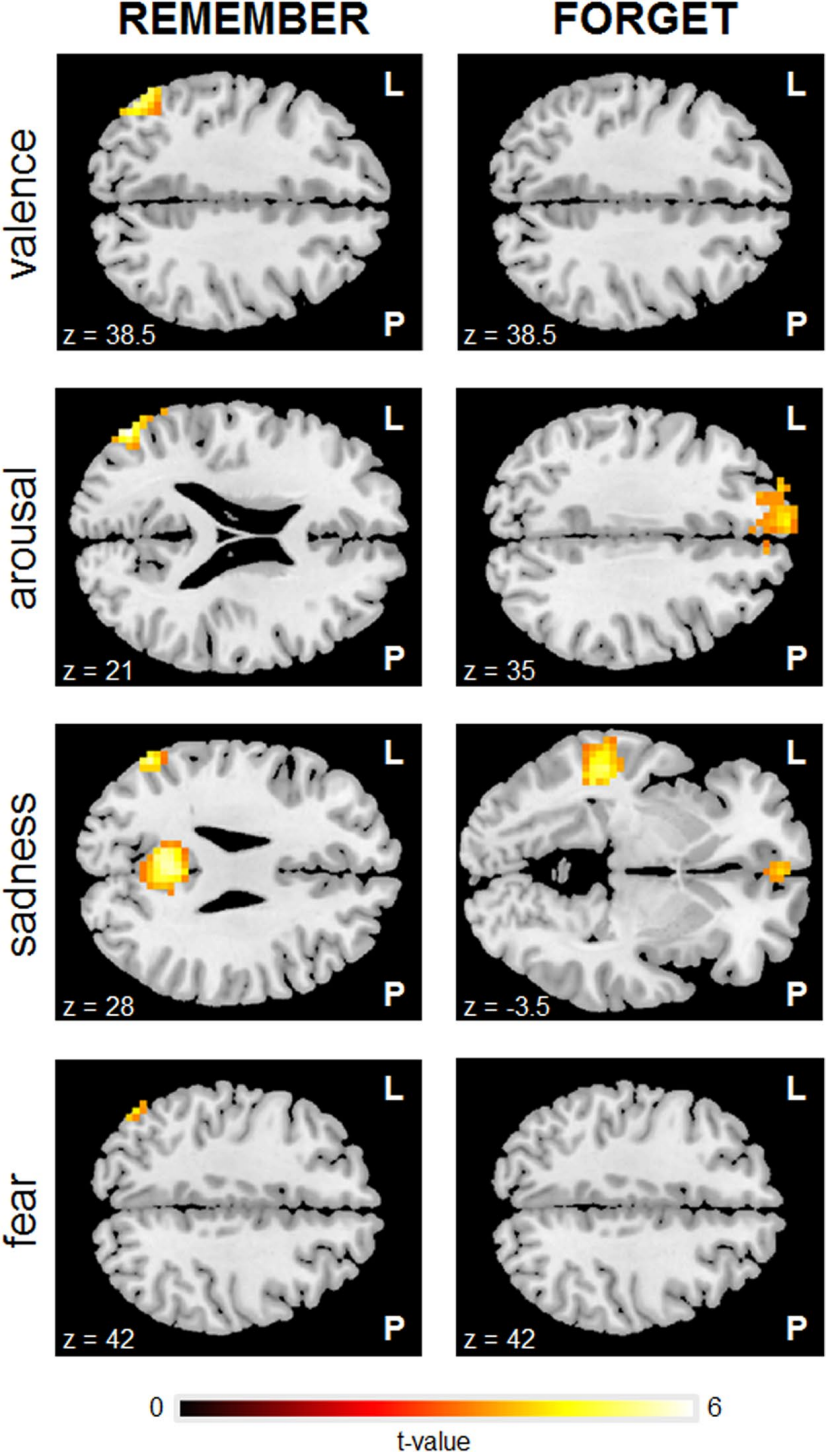
Whole-brain statistical parametric maps representing task-related brain activation correlated with behavioural parameters (VAL – valence, ARO – arousal, SAD – sadness, FEA – fear) during encoding (TBR) and encoding suppression (TBF). None of the parameters modulated the activity in the lateral PFC, suggesting that multiple regions are likely to play a role in the cognitive control mechanisms. A voxel-wise height threshold of *p* < 0.001 (uncorrected) combined with a cluster-level extent threshold of *p* < 0.05 (corrected for multiple comparisons using the FWE rate) was applied.

All tested parameters (valence, arousal, sadness, fear) were found to modulate task-related activity. The patterns of parametrically modulated brain activity during encoding attempt (TBR > baseline) were quite similar for all tested parameters and revealed significant effects mainly located in left lateral occipital cortex, left angular gyrus and left supramarginal gyrus. None of the parameters were found to modulate the activity in the right lateral prefrontal regions during encoding suppression (TBF > baseline). Interestingly, arousal and sadness were the only parameters exhibiting any suprathresholded modulation of the activity during encoding suppression. For both parameters, these effects included the activity in medial aspect of the frontal pole. Detailed results are presented in Table 5 and Fig. 6.

**Table 5:** Peak level activations for the parametric modulations analysis. Notation: TBR – to-be-remembered items, TBF – to-be-forgotten items, VAL – valence, ARO – arousal, SAD – sadness, FEA - fear. A voxel-wise height threshold of *p* < 0.001 (uncorrected) combined with a cluster-level extent threshold of *p* < 0.05 (corrected for multiple comparisons using the FWE rate) was applied. Activations surviving a peak-level FWE-corrected threshold of p < 0.05 are marked with an asterisk. Table shows all local maxima separated by more than 8 mm. Regions were automatically labelled using the Harvard-Oxford atlas. x, y, and z Montreal Neurological Institute (MNI) coordinates in the left-right, anterior-posterior, and inferior-superior dimensions, respectively.

Harvard-Oxford label of the peak	Cluster extent	Hemisphere	t-value	MNI coordinates			*p*_FWE_
				x	y	z	
VAL * TBR							
Lateral Occipital Cortex, superior division	75	L	5.218	−49	−67	39	**0**.020*
Lateral Occipital Cortex, superior division		L	5.105	−56	−63	35	**0**.029*
VAL * TBF							
*no supratreshold clusters*							
ARO * TBR							
Lateral Occipital Cortex superior division	74	L	5.920	−53	−70	21	**0**.001*
Lateral Occipital Cortex superior division		L	4.296	−42	−77	32	0.347
Supramarginal Gyrus posterior division		L	3.968	−63	−53	25	0.665
ARO * TBF							
Frontal Pole	215	L	4.421	−7	60	35	0.253
Frontal Pole		L	4.282	−11	67	28	0.358
Frontal Pole		L	3.994	−25	56	35	0.639
SAD * TBR							
Cingulate Gyrus posterior division	185	L	5.854	0	−53	32	**0**.002*
Angular Gyrus	54	L	5.317	−56	−60	28	**0**.018*
Lateral Occipital Cortex superior division		L	4.512	−46	−74	39	0.225
SAD * TBF							
Middle Temporal Gyrus posterior division	222	L	5.055	−53	−32	−4	**0**.044*
Middle Temporal Gyrus posterior division		L	4.822	−49	−18	−11	0.092
Supramarginal Gyrus posterior division		L	4.198	−63	−42	7	0.478
Frontal Pole	108	L	4.468	−4	56	39	0.253
Frontal Pole		L	4.357	−4	60	4	0.335
Frontal Pole		L	4.151	−7	60	14	0.525
FEA * TBR							
Lateral Occipital Cortex, superior division	70	L	4.292	−49	−67	42	0.357
Lateral Occipital Cortex, superior division		L	4.226	−53	−67	32	0.415
Lateral Occipital Cortex, superior division		L	3.906	−46	−74	39	0.728
FEA * TBF							
*no supratreshold clusters*

### Functional connectivity results

**Figure 7:**
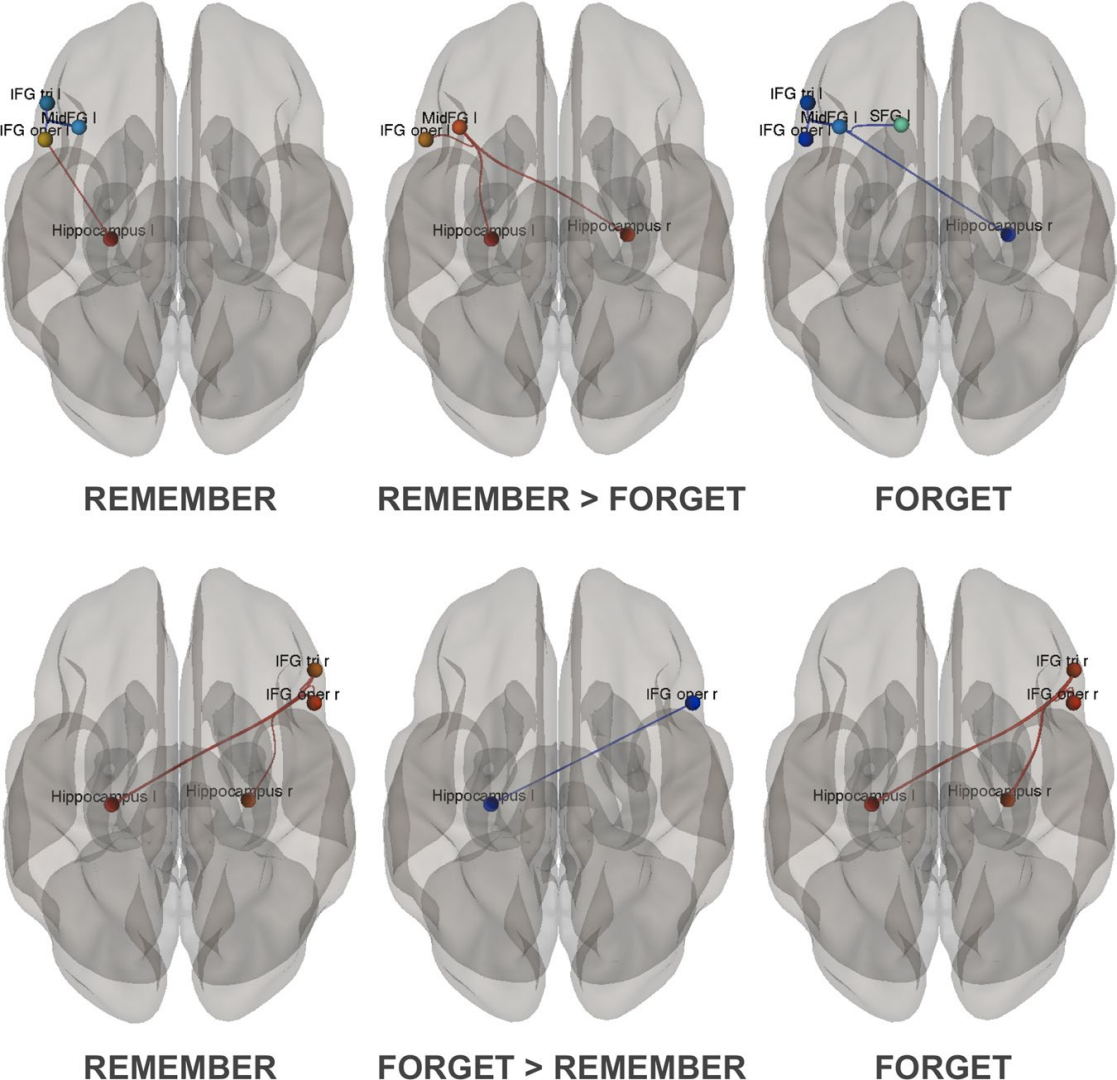
Changes in the task-related ROI-to-ROI functional connectivity between the lateral prefrontal ROIs and the hippocampi specific to encoding (TBR > TBF) and encoding suppression (TBF > TBR) conditions (p < 0.05, FDR). Red color indicates increase, whereas blue color indicates decrease in task-related correlation between respective ROIs. Comparisons to the baseline (TBR > baseline, TBF > baseline) are also presented for illustrative purposes. Red color indicates positive correlation, whereas blue color indicates negative correlation between respective ROIs.

We observed changes in the ROI-to-ROI connectivity among the lateral prefrontal cortex and the hippocampi (Fig. 7). In particular, we obtained a significant increase in connectivity between left MidFG and left hippocampus (T(24) = 2.34; *p*_*unc*_ = 0.0280; *p*_*FDR*_ = 0.0470), as well as right hippocampus (T(24) = 2.63; *p*_*unc*_ = 0.0148; *p*_*FDR*_ = 0.0470) related to encoding attempt (TBR > TBF). On the other hand, connectivity between right opercular part of IFG and left hippocampus (T(24) = −2.82; *p*_*unc*_ = 0.0095; *p*_*FDR*_ = 0.0473) was decreased for encoding suppression (TBF > TBR). We were unable to observe similar effects in comparisons performed separately for neutral (NEU TBR – NEU TBF) or emotional (EMO TBR – EMO TBF) trials.

Finally, changes in the ROI-to-ROI connectivity among the lateral prefrontal cortex and the VWFA were found neither during encoding attempt (TBR > TBF), nor during encoding suppression (TBF > TBR). Similarly, we were unable to demonstrate any effects specific to neural or emotional words.

## Discussion

Previous research has already shown that cognitive control over memory can affect not only retention, but also suppression of memories^1^. While the specific role of the lateral prefrontal cortex (lateral PFC) in these cognitive control mechanisms was widely confirmed, most recent findings highlight that the contribution of multiple regions is more likely to play a role^17,18^. In particular, previous studies demonstrated that during memory suppression the lateral PFC is responsible for the regulation of hippocampus, as well as other regions involved in the reinstatement of sensory features of memory representations^15^. Similarly, attempts were made to investigate whether the lateral PFC regulates the activity of regions involved in the processing of emotional features of memory traces^28^. Claims that the engagement of the lateral PFC depends on emotion have thus far been inconsistent^9,26–28^. As recently pointed out, these inconsistencies might be due to the fact that individuals differ in their memory performance^28^. The present study sought to clarify whether emotion influences subjects’ ability to control memory. Thus, we used the *item-wise directed forgetting* paradigm to measure the individual level of cognitive control over encoding of neutral and emotional (negatively valenced) words. Moreover, we collected subjects’ individual ratings of the words on scales of valence, arousal, sadness and fear. We provided behavioural evidence from two independent samples (*non-fMRI* and *fMRI*), to assess the stability of the reported results. Furthermore, we demonstrated the pattern of neuronal activity, as well as functional connectivity corresponding to the discussed behavioural effects.

### Role of the lateral PFC in encoding and its inhibition/suppression

As expected, we observed a behavioural effect of directed forgetting, that is better memory performance for to-be-remembered (TBR) than for to-be-forgotten (TBF) words. Specifically, we found that the proportion of correct responses depended on the instruction type. Moreover, the instruction effect was still present when we accounted for both hits and false alarms, and compared the corresponding d’ values. We were able to replicate this effect in two independent samples that completed the task in different experimental settings (outside and inside the MRI machine). Combined analysis of both samples demonstrated the stability of the results. Furthermore, in each sample the directed forgetting effect was found irrespective of the stimulus material. Similarly, the directed forgetting effect has already been demonstrated for various types of experimental stimuli^1,8–11,26,27,35^.

As hypothesized, at the neuronal level we found the left lateral prefrontal areas to be specifically involved in encoding effort, whereas the right lateral prefrontal areas in the suppression of encoding. Specifically, we found the triangular part of the left IFG, as well as the left MidFG and SFG to be more active when subjects were instructed to remember words, while the right MidFG and the right SFG to be more active when they were instructed to forget words. Likewise, with the use of various experimental paradigms (i.e. *item-wise directed forgetting*, *list-wise directed forgetting*, *think/no-think*) previous research repeatedly found the left lateral prefrontal activity to be related to encoding or retrieval, while the right lateral prefrontal activity to their inhibition/suppression^1^. Furthermore, our findings seem to confirm previous reports on the role of the right MidFG in memory inhibition^14^. However, we did not observe any involvement of the right IFG, a region most directly related to behavioural inhibition^12,13^. Importantly, apart from the lateral PFC, we found multiple other brain regions related to task performance, many of which are commonly associated with the frontoparietal control network^20,21^. Furthermore, we explored the underlying functional connectivity. In line with our expectations, we found that encoding attempt was associated with increased connectivity between the left MidFG and the left and right hippocampi, whereas encoding suppression was accompanied by decreased connectivity between the right IFG and the left hippocampus. Our results are consistent with previous reports on the functional connectivity changes related to cognitive control over memory during encoding^35^ and retrieval^4,14^. Importantly, these studies used different methods to assess memory performance, demonstrating that interactions between the lateral PFC and the hippocampus were crucial for subsequent memory performance, no matter whether the items were recognized^35^ or recalled^4,14^. In particular, these studies demonstrated that increased activity in the right dorsolateral prefrontal cortex during memory suppression predicted decreased activity in the hippocampal cortex^4,14^. We did not detect changes in functional connectivity between the lateral prefrontal regions and the visual word form area (VWFA). However, it might be the case that our attempt to localize VWFA based on previously reported coordinates was imprecise and its prior identification with functional localizer procedure would yield different results. In fact, with the use of nonverbal material (images)^15^ demonstrated that suppression mechanisms mediated by the right MidFG modulated the activity in other regions hypothesized to participate in memory formation process, i.e. visual cortex or fusiform cortex.

### Individual differences in memory performance for emotional and neutral material likely contribute to the lateral PFC engagement

In line with previous research, we observed the directed forgetting effect irrespective of emotion category. This effect has already been demonstrated for neutral, as well as emotional images^9,26^ and words^27^. On the other hand, we found that instruction had on average smaller impact on memory performance in the case of emotional, as compared to neutral words. Similarly^9,27^, found the directed forgetting to be weaker for emotional stimuli. Yet, in the present study we observed rather small effect owing to emotion, especially when the emotion categories (neutral, fear and sadness) were compared. Thus, our results do not confirm previous report of^26^, who studied memory using diverse emotional images (i.e. related to sadness, fear or disgust) and found differences in the strength of the directed forgetting effect. Furthermore, we found that the proportion of false alarms was on average higher for emotional, as compared to neutral words. In fact, difference in the directed forgetting effect owing to emotion was no longer present when we accounted for both: difference in hits, as well as difference in false alarms. However^28^, recently emphasized the role of individual differences in the ability to suppress memory. Following this line of research, we included individual ratings of valence, arousal, sadness and fear and individual task performance in our subsequent analysis of the relation between individual ratings and memory performance. We showed that emotion influenced task performance in opposite ways depending on trial type: improved performance for studied, old items (higher scores of VAL, ARO, SAD, FEA for words in the set of correct recognitions) and impaired performance for unstudied, new items (higher scores of VAL, ARO, SAD, FEA for words in the set of false alarms).

Brain activity patterns corresponding to encoding attempt and encoding suppression were further explored in relation to emotion. Although we found the instruction effects to more strongly engage the activity of the lateral PFC during emotional, as compared to neutral trials, direct comparisons between the two revealed no significant difference. Recent study by^27^ showed the right MidFG to be more active during encoding suppression of neutral as compared to negative words. Based on these results^27^, concluded that emotion disrupted inhibitory control, presumably due to more cognitive resources being directed to negative stimuli. On the other hand, an earlier study by^9^ found the activation of the right MidFG corresponding to encoding suppression to be much stronger for negative images than for neutral ones. These results were interpreted by^9^ as reflecting more cognitive resources being engaged in the suppression of negative images. Contrary to previous reports, we observed neither more, nor less engagement of the lateral PFC during encoding or encoding suppression of emotional (as compared to neutral) material. Furthermore, our subsequent ROI analyses also failed to reveal significant differences in the contrast estimate values between neutral and emotion conditions. Finally, we found no evidence for the claim that the connectivity patterns between the lateral PFC regions and regions hypothesized as involved in the memory formation process (i.e. hippocampal cortex, fusiform cortex) are influenced by emotion.

Recent findings emphasized that people differ in their ability to suppress memory^28^. Therefore in the present study we examined whether task-related lateral PFC engagement depended on subjects’ task performance. We controlled for individual differences in two ways: by using individual memory performance, and by using individual behavioural ratings of valence, arousal, sadness and fear. Having accounted for individual differences in memory performance, we were able to demonstrate that the lateral PFC engagement depended on subjects’ cognitive abilities to control their memory, as measured by the DF effect. Moreover, we showed that this dependence is specific to emotional memories, in contrast to neutral ones. This result resembles a recent report of^28^, who emphasized the role of individual differences in memory suppression. In particular^28^, found that cognitive effort to suppress retrieval can result in reduced access to both mnemonic and emotional content. Specifically, the increase in the activity of the right MidFG predicted fewer intrusions and reduced affect. On the contrary, the decrease in the activity of the amygdala, hippocampus and parahippocampal cortex predicted fewer intrusions and reduced affect^28^. Having accounted for individual differences in the ratings of valence, arousal, sadness and fear, we showed that task-related brain activity depended on the emotional properties of the memorized material. Yet, none of the emotional parameters (valence, arousal, sadness, fear) modulated the activity in the lateral PFC during the encoding or encoding suppression. During the encoding attempt, all parameters produced similar effects, revealing parametrically modulated brain activity, mainly located in the left lateral occipital cortex, left angular gyrus and left supramarginal gyrus, often referred to as the inferior parietal lobule (IPL). Interestingly, a recent review by^21^ highlighted the crucial role of the IPL, a highly connected region implicated in a broad range of higher cognitive functions. As the IPL was found to belong to multiple functional networks, it is plausible that this region plays an important role in communication between different networks, as well as in integration of information, crucial to higher-order cognition. During encoding suppression, we identified the medial aspect of the left frontal pole to be parametrically modulated, but only by the arousal and sadness parameters. In a recent large-scale meta-analysis^36^ demonstrated that the dorsal middle cingulate cortex (dorsal MCC) and the dorsal medial prefrontal cortex (dmPFC) coactivates most strongly with the regions belonging to the frontoparietal control network. While our results indicate the importance of individual differences in cognitive control over memory, it is more likely that multiple regions contribute to this effect.

### Concluding remarks and future directions

Previous research on cognitive control over memory emphasized the role of the lateral prefrontal cortex, neglecting the contribution of other regions^20,21^. The present study suggests that the cognitive mechanisms related to control over memory are supported by a network of regions, rather than any single structure. Future studies should focus on the way in which cognitive processes underlying control over memory are reflected in interactions within a network of regions. The involvement of multiple brain regions is especially perplexing when there is no consensus for what cognitive processes underlie control over memory^17,19^. Future studies should attempt to disentangle multiple cognitive mechanisms that might be involved in the performance of tasks used to study memory suppression, such as *directed forgetting* or *think/no-think*. Similarly, individual differences should be taken into account in order to fully understand brain mechanisms underlying cognitive control in memory. Only when these aspects are carefully controlled, it may be possible to determine whether and how emotion influences control over memory.

### Data availability

The datasets generated during and/or analyzed during the current study are available from the corresponding author upon request.

## References

[1] Anderson MC, Hanslmayr S (2014). Neural mechanisms of motivated forgetting. Trends Cogn. Sci..

[2] Baddeley, A., Eysenck, M. W. & Anderson, M. C. *MEMORY*. (Psychology Press, Taylor & Francis Group, 2015).

[3] Hasson U, Chen J, Honey CJ (2015). Hierarchical process memory: memory as an integral component of information processing. Trends Cogn. Sci..

[4] Benoit RG, Anderson MC (2012). Opposing mechanisms support the voluntary forgetting of unwanted memories. Neuron.

[5] Bjork RA (1970). Positive forgetting: The noninterference of Items intentionally forgotten. J. Verbal Learning Verbal Behav..

[6] Bjork, R. A. Retrieval Inhibition as an Adaptive Mechanism in Human Memory. *Var*. *Mem*. *Conscious*. 309–330 (1989).

[7] Anderson MC, Green C (2001). Suppressing unwanted memories by executive control. Nature.

[8] Bastin C (2012). The Neural Substrates of Memory Suppression: A fMRI Exploration of Directed Forgetting. PLoS One.

[9] Nowicka A, Marchewka A, Jednorog K, Tacikowski P, Brechmann A (2011). Forgetting of Emotional Information Is Hard: An fMRI Study of Directed Forgetting. Cereb. Cortex.

[10] Reber PJ (2002). Neural correlates of successful encoding identified using functional magnetic resonance imaging. J. Neurosci..

[11] Wylie GR, Foxe JJ, Taylor TL (2008). Forgetting as an Active Process: An fMRI Investigation of Item-Method-Directed Forgetting. Cereb. Cortex.

[12] Aron AR, Robbins TW, Poldrack RA (2004). Inhibition and the right inferior frontal cortex. Trends Cogn. Sci..

[13] Aron AR, Robbins TW, Poldrack RA (2014). Inhibition and the right inferior frontal cortex: one decade on. Trends Cogn. Sci..

[14] Depue BE, Orr JM, Smolker HR, Naaz F, Banich MT (2016). The Organization of Right Prefrontal Networks Reveals Common Mechanisms of Inhibitory Regulation Across Cognitive, Emotional, and Motor Processes. Cereb. Cortex.

[15] Gagnepain P, Henson RN, Anderson MC (2014). Suppressing unwanted memories reduces their unconscious influence via targeted cortical inhibition. Proc. Natl. Acad. Sci. USA.

[16] Anderson MC (2004). Neural Systems Underlying the Suppression of Unwanted Memories. Science (80-.)..

[17] Erika-Florence M, Leech R, Hampshire A (2014). A functional network perspective on response inhibition and attentional control. Nat. Commun..

[18] Hampshire A, Sharp DJ (2015). Contrasting network and modular perspectives on inhibitory control. Trends Cogn. Sci..

[19] Friedman NP, Miyake A (2017). Unity and diversity of executive functions: Individual differences as a window on cognitive structure. Cortex.

[20] Vincent JL, Kahn I, Snyder AZ, Raichle ME, Buckner RL (2008). Evidence for a Frontoparietal Control System Revealed by Intrinsic Functional Connectivity. J. Neurophysiol..

[21] Igelström, K. M. & Graziano, M. S. A. The inferior parietal lobule and temporoparietal junction: A network perspective. *Neuropsychologia* 1–14, 10.1016/j.neuropsychologia.2017.01.001 (2017). 10.1016/j.neuropsychologia.2017.01.001 28057458

[22] Cole DM, Smith SM, Beckmann CF (2010). Advances and pitfalls in the analysis and interpretation of resting-state FMRI data. Front. Syst. Neurosci..

[23] Bressler SL, Menon V (2010). Large-scale brain networks in cognition: emerging methods and principles. Trends Cogn. Sci..

[24] Pessoa L (2009). How do emotion and motivation direct executive control?. Trends Cogn. Sci..

[25] Pessoa, L. *The cognitive-emotional brain*. *From interactions to integration*. (The MIT Press, 2013).

[26] Marchewka A (2016). What Is the Effect of Basic Emotions on Directed Forgetting? Investigating the Role of Basic Emotions inMemory. Front. Hum. Neurosci..

[27] Yang T, Lei X, Anderson MC (2016). Decreased inhibitory control of negative information in directed forgetting. Int. J. Psychophysiol..

[28] Gagnepain, P., Hulbert, J. & Anderson, M. C. Parallel Regulation of Memory and Emotion Supports the Suppression of Intrusive Memories. *J*. *Neurosci*. at http://www.jneurosci.org/content/early/2017/05/30/JNEUROSCI.2732-16.2017 (2017). 10.1523/JNEUROSCI.2732-16.2017 PMC5511877 28559378

[29] Benoit, R. G., Davies, D. J. & Anderson, M. C. Reducing future fears by suppressing the brain mechanisms underlying episodic simulation. *Proc*. *Natl*. *Acad*. *Sci*. *USA* **113**, E8492–E8501 (2016). 10.1073/pnas.1606604114 PMC5206570 27965391

[30] McCandliss BD, Cohen L, Dehaene S (2003). The visual word form area: expertise for reading in the fusiform gyrus. Trends Cogn. Sci..

[31] Dehaene S, Cohen L (2011). The unique role of the visual word form area in reading. Trends Cogn. Sci..

[32] Riegel M (2015). Nencki Affective Word List (NAWL): the cultural adaptation of the Berlin Affective Word List–Reloaded (BAWL-R) for Polish. Behav. Res. Methods.

[33] Wierzba M (2015). Basic Emotions in the Nencki Affective Word List (NAWL BE): New Method of Classifying Emotional Stimuli. PLoS One.

[34] Wickens, T. D. *Elementary signal detection theory*. (Oxford University Press, 2002).

[35] Rizio AA, Dennis NA (2013). The Neural Correlates of CognitiveControl: Successful Remembering and Intentional Forgetting. J. Cogn. Neurosci..

[36] de la Vega A, Chang LJ, Banich MT, Wager TD, Yarkoni T (2016). Large-Scale Meta-Analysis of Human Medial Frontal Cortex Reveals Tripartite Functional Organization. J. Neurosci..

